# Utility of Computational Models in Predicting the Efficacy of Stem Cell Therapy for Schizophrenia – Insights from a Systematic Review and Meta-Analysis

**DOI:** 10.1192/j.eurpsy.2025.2280

**Published:** 2025-08-26

**Authors:** J. Parmar, A. A. Pillai, R. Walwaikar, A. Agrawal, A. A. Kumar, A. S. Nagendrapandian, F. Sheikh

**Affiliations:** 1 Government Medical College, Amritsar; 2 SSPM Medical College and Lifetime Hospital, Sindhudurg, India; 3 Humanitas University, Milan, Italy; 4 GMERS Medical College, Vadnagar, India; 5 West Windsor Plainsboro High School South, New Jersey, United States; 6 Greater manchester mental health trust, Manchester, United Kingdom

## Abstract

**Introduction:**

Schizophrenia is a disorder associated with significant morbidity, largely due to poor treatment outcomes with existing interventions. Emerging evidence demonstrates that stem cell therapy using either patient-derived induced pluripotent stem cells (iPSCs) or mesenchymal stem cells (MSCs), may offer an effective alternative for treating schizophrenia by promoting the restoration of excitatory interneurons. However, given the variability in the therapeutic potential of iPSCs and MSCs, adopting a progressive computational approach to predict the clinical outcomes of these therapies might be an effective strategy.

**Objectives:**

The objective was to evaluate the efficacy of stem cell therapy in schizophrenia and to explore the role of computational models in predicting the outcomes of this therapy.

**Methods:**

We conducted a systematic search of clinical trials and studies published (since 2015) in PubMed, SCOPUS, and EMBASE. The review included all randomized controlled trials involving iPSC or MSCs-based interventions and studies that incorporated computational models to predict outcomes. A total of 22 studies including 1436 individuals were included in the review. Meta-analytic methods were used to calculate pooled effect sizes on cognitive outcomes and reduction or improvement in negative symptoms was recorded using standardized mean difference (SMD) and risk ratios (RR).

**Results:**

This involved 979 patients with schizophrenia from four studies that met quality review criteria, revealing that MSC-based therapies using positive controls significantly improved negative symptoms with a standardized mean difference (SMD) of 0.52 (95% CI, 0.32–0.73; *P* < 0.001). Improvements in cognition, especially in the domains of memory and executive function, were significant in treated groups using iPSCs (SMD = 0.61, 95% CI, 0.40–0.82; *P* < 0.0001). The predictive models that classified interneuron (PV and SST) restoration in terms of sensitivity (83.4%) and specificity (78.2%) enhanced the ability to predict responder treatment effects. Ultimately, computational modeling reduced predictive variance in therapeutic efficacy by 18.7% (p = 0.006).

**Conclusions:**

Our meta-analysis revealed that stem cell therapies, particularly MSCs and iPSCs, significantly improved both negative and cognitive symptoms associated with schizophrenia. Additionally, predictive models using computational methods were found to accurately predict the therapeutic outcomes for intervention treatments based on the resting patient subgroups that received interneuron restorations. We conclude that stem-cell-based therapies especially when used alongside computational models have tremendous potential to provide precise and personalized psychiatric care.

**Disclosure of Interest:**

None Declared

